# Japanese cancer survivors have a higher risk of fragility fractures over ten years

**DOI:** 10.1038/s41598-026-45389-1

**Published:** 2026-03-23

**Authors:** Takaomi Kobayashi, Yuichiro Nishida, Takuma Furukawa, Chisato Shimanoe, Mikako Horita, Hinako Nanri, Yasuki Higaki, Tadatsugu Morimoto, Keitaro Tanaka, Megumi Hara

**Affiliations:** 1 Department of Preventive Medicine, Faculty of Medicine, Saga University, 5-1-1, Nabeshima, Saga, 849-8501 Japan; 2 Department of Orthopaedic Surgery, Faculty of Medicine, Saga University, 5-1-1 Nabeshima, Saga, 849-8501 Japan; 3 Department of Liberal Arts, Faculty of Healthcare and Welfare, Saitama Prefectural University, 820 Sannomiya, Koshigaya-shi, Saitama, 343-8540 Japan; 4 Education and Research Center for Community Medicine, Faculty of Medicine, Saga University, 5-1-1, Nabeshima, Saga, 849-8501 Japan; 5 Department of Pharmacy, Saga University Hospital, 5-1-1 Nabeshima, Saga, 849-8501 Japan; 6 Laboratory of Behavioral Physiology, Center for Clinical Nutrition, National Institutes of Health and Nutrition, Health and Nutrition, National Institutes of Biomedical Innovation, Kento Innovation Park, NK Building, 3-17 Senrioka Shinmachi, Settsu-shi, 566- 0002 Osaka Japan; 7 Laboratory of Exercise Physiology, Faculty of Sports and Health Science, Fukuoka University, Nakamura 8-19-1 Jyonan-ku, Fukuoka, 814-0180 Japan

**Keywords:** Cancer, Fracture, Japanese, Time-dependent variable, Prevention, Cancer, Oncology, Risk factors

## Abstract

This study aimed to clarify the risk of fragility fractures (FFs) in Japanese cancer survivors compared with individuals without cancer by incorporating time-dependent variables. In a 10-year cohort of 10,330 adults aged 40–69 years, we assessed the risk of FFs (i.e., hip, vertebral compression, and distal radius fractures) associated with cancer status by using time-dependent Cox proportional hazards analyses. The models were adjusted for demographic, lifestyle, and medical factors. The time-dependent variables included cancer status and age. Analyses were performed according to cancer treatment status, number of primary cancers, and cancer site. Among the 10,330 participants (mean age, 56.4 years; females, 6148), 386 experienced FFs. Compared with participants without cancer, a higher risk of overall FFs was observed in participants with cancer (hazard ratio [HR], 1.40; 95% confidence interval [CI], 1.03–1.89). This risk was further elevated among participants with active cancer (HR, 1.71; 95% CI, 1.15–2.55), multiple primary cancers (HR, 1.42; 95% CI, 1.04–1.95), stomach cancer (HR, 1.84; 95% CI, 1.01–3.37), kidney cancer (HR, 3.74; 95% CI, 1.20–11.70), and hematologic cancer (HR, 7.70; 95% CI, 2.45–24.16). These findings highlight the importance of FF prevention strategies in cancer survivorship care in aging Asian populations.

**Supplementary Information:**

The online version contains supplementary material available at 10.1038/s41598-026-45389-1.

## Introduction

As the world’s first super-aged society, Japan has entered the “cancer era,” wherein improvements in cancer survival rates have made the maintenance of motor function increasingly important in cancer care^1^. Cancer survivors are at an increased risk of fragility fractures (FFs) owing to various factors, including direct musculoskeletal damage from cancer, treatment-related effects, musculoskeletal comorbidities, and cancer-related complications^1,2^. In support of this concern, large epidemiological studies have reported an elevated risk of FFs in cancer survivors in a US cohort of older adults^3^, population-based matched cohort studies in the United Kingdom^4^ and Canada^5^, and a case–control study in Denmark^6^. Although these studies offer valuable insights, their findings do not provide answers to several important questions.

While many previous studies have focused on specific cancer types or treatment-related fracture^7–20^, epidemiological evidence capturing the overall FF risk among cancer survivors in the general population remains limited, particularly in Asian cohorts. Furthermore, certain methodological considerations may have influenced the risk estimation. For example, the exclusion of the pre-diagnosis period of cancer in cohort studies could have led to an overestimation of fracture risk^3^ because fractures occurring before cancer diagnosis would be excluded from the person-time of cancer survivors. Similarly, case–control designs might have introduced complexities related to participant selection, potentially distorting the observed associations owing to differences in baseline characteristics between cases and controls^4–6^. Time-dependent Cox proportional hazards analysis, which has been increasingly discussed in recent epidemiological research, may help address one of these considerations, particularly the arbitrary exclusion of observation periods in cohort studies, by accounting for the pre-diagnosis period and providing more accurate risk estimates^21,22^.

In addition to methodological concerns, the current evidence remains limited with respect to treatment status, multiple primary cancers, site-specific fractures, and bone metastases. Current therapies such as surgery, radiation, chemotherapy, and hormone deprivation therapy, which are used across various cancers^7–20,23–37^, could increase the fracture risk more than previous therapies. Among survivors with multiple primary cancers (2%–17%^38^, the cumulative effects of disease and treatment could reduce bone mineral density and increase the risk of FFs. Distal radius fractures are common in active middle-aged individuals in the general population^39,40^; therefore, the prevalence of such fractures in cancer survivors may be similarly elevated. Bone metastases, which often involve fracture-prone sites^3,41^, were rarely excluded in previous studies; however, even non-metastatic cancers could increase the risk of FFs via systemic or treatment-related effects.

This study aimed to clarify the risk of FFs, including hip, vertebral compression, and distal radius fractures, among Asian cancer survivors by incorporating time-dependent variables. The secondary aim was to examine the risk of FFs in individuals with cancer according to cancer treatment status, number of primary cancers, and cancer site in comparison with those of individuals without cancer.

## Methods

### Study design

We conducted a prospective cohort study involving participants from the Japan Multi-Institutional Collaborative Cohort (J-MICC) Study conducted in Saga Prefecture. The baseline survey (2005–2007) has been reported previously^42–44^. In brief, 61,447 residents aged 40–69 years in Saga City were invited to participate via mail, and surveys were conducted by telephone. A total of 12,078 individuals participated in the baseline survey. In the 10-year follow-up survey (2015–2017), a validated questionnaire^45^ was mailed to all participants 10 years after the baseline survey to collect self-reported information on fracture history and location over the past 10 years. There was only one reminder, and inquiries regarding omissions were made via phone. Overall, 10,332 individuals participated in the 10-year follow-up survey. Information on self-reported cancer diagnoses was collected during the baseline survey. Among the 10,332 participants, those with metastatic cancers (liver and breast) at the time of the baseline survey (*n* = 2) were excluded. Ultimately, 10,330 participants were included in the analysis.

In the baseline survey of the J-MICC study, informed consent was obtained from participants for a follow-up survey via mail on the incidence of various diseases. In addition, participants were asked to complete a follow-up survey via mail regarding fractures, and their responses were considered as consent to participate in the follow-up surveys in each field.

This study was approved by the Institutional Review Board of Saga University School of Medicine (approval no. 17 − 11) and Nagoya University Graduate School of Medicine (approval no. 253), and was conducted in accordance with the Declaration of Helsinki. Regarding the description of this study, the Strengthening the Reporting of Observational Studies in Epidemiology (STROBE) guidelines were followed^46^.

### Cancers

All cancer information before the baseline survey was assessed by self-reporting and was not validated in this cohort, whereas self-reported cancers during the follow-up period were confirmed by medical record review.

Treatment status was assessed as follows: cancer after therapy (i.e., previous cancer diagnosis), which refers to participants who are now cured of cancer but have had cancer in the past (including those who are being monitored after recovery), and cancer under therapy (i.e., active cancer), which refers to participants who currently have cancer or are undergoing treatment (including follow-up before treatment begins).

The number of primary cancers in individuals was assessed as single primary cancer or multiple primary cancers. Multiple primary cancers involve multiple malignancies that develop in different tissues with distinct morphologies.

On the basis of the theoretical relationship reported thus far^7–20,23–38^, the cancer sites of interest were set as follows: stomach cancer, colon and rectal cancer, thyroid cancer, bladder cancer, kidney cancer, hematologic cancer (leukemia, lymphoma, or myeloma), breast cancer, gynecological cancer (uterine and ovarian cancer), and prostate cancer. The cancer types that were not of interest were defined as other cancers. For individuals with multiple primary cancers, the site of first cancer diagnosis was used.

### Fractures

All self-reported FFs during the follow-up period were validated by medical record review in the Saga cohort of the J-MICC Study^45^. During the 10-year follow-up survey, study participants were asked for information regarding fractures, the number of years from the baseline survey to the fractures, and the origin of the injury experienced in the past 10 years (i.e., minor falls, sports, traffic accidents, and others^45^. The current study focused on FFs due to minor falls, including hip, vertebral compression, and distal radius fractures, as these sites are conventionally defined as major FFs in previous epidemiological studies^45,47^. Fractures of the proximal humerus and pelvis were not included because these fracture sites could not be identified from the questionnaire^45^, and these fracture sites are not consistently categorized as FFs in previous large-scale epidemiological studies^45,47^. Vertebral compression fractures were defined as those accompanied by acute pain and confirmed radiographically. When analyzing the number of years from the baseline survey to the FFs, the number of years until the first fracture among hip, vertebral compression, or distal radius fractures was used.

### Covariates

Data on potential confounding factors were collected from the baseline survey. The covariates included sex, menopausal state, age, body mass index (BMI), highest educational attainment, number of comorbidities, history of hormone replacement therapy, osteoporosis medication use, corticosteroid use, smoking status, alcohol consumption, and physical activity, all of which (except for BMI) were assessed using a self-reported questionnaire^42–45^. Although completeness was confirmed at the time of data collection, a small number of missing values were subsequently identified. These were supplemented using follow-up survey responses for time-invariant and lifestyle variables assessed through habitual or lifetime history questions, resulting in a complete analytical dataset.

Regarding menopausal status, participants who answered the question “Is your menstruation continuing?” with “still ongoing,” “gradually stopping,” or “stopped” were classified as premenopausal, perimenopausal, or postmenopausal, respectively. Educational attainment was assessed using the question “What is the highest level of education you have completed?” Responses were categorized as follows: “elementary or junior high school” as below high school; “high school” as high school; and “vocational school,” “junior college,” “technical college,” “university,” and “graduate school” as college or trade school. The comorbidities included emphysema or chronic bronchitis, stroke or transient ischemic attack, heart attack or coronary artery disease, high cholesterol, hypertension, and type 2 diabetes. On the basis of the total number of comorbidities reported, the participants were grouped into four categories: zero, one, two, and three or more comorbidities^3^. History of hormone replacement therapy use was defined as an answer of “yes” to the question “Have you ever used hormone medication for more than four weeks (one month) in total, for reasons such as menopausal symptoms, contraception, infertility, or treatment following ovarian surgery?” Osteoporosis treatment was defined as the regular use (at least once per week) of osteoporosis medication (e.g., bisphosphonates, selective estrogen receptor modulators, and/or vitamin D), and participants were classified as treated or untreated. Similarly, participants who reported the regular corticosteroid use (at least once per week) were classified as corticosteroid users, and those who did not were considered non-users. Smoking status was based on responses to the question “Do you smoke?” Participants were classified as non-smokers, past smokers, or current smokers if they responded with “I do not smoke,” “I quit,” or “I smoke,” respectively. Current smokers were further classified according to the number of cigarettes smoked per day into light (≤ 9 cigarettes/day), moderate (10–19 cigarettes/day), or heavy smokers (≥ 20 cigarettes/day). We used a validated short food frequency questionnaire^48^ to estimate alcohol consumption and total energy intake. Alcohol consumption was calculated in units/day (20 g was calculated as one unit)^47^. Alcohol consumption was categorized as none (zero units/day), light (one to two units/day), or moderate/heavy (three or more units/day). The total energy intake (kcal/day) was calculated using a program developed by the Department of Public Health, Nagoya City University School of Medicine, mainly on the basis of the intake of energy-producing nutrients such as carbohydrates (4 kcal/g), lipids (9 kcal/g), proteins (4 kcal/g), and alcohol (7 kcal/g)^49^. In the present study, physical activity was calculated by summing all daily and leisure time activities on the basis of a questionnaire similar to the one with proven validity and reliability used in the Japan Public Health Center–based prospective study^50^. Physical activity was categorized as low (< 10 metabolic equivalent [MET] hours/week), middle (≥ 10 and < 50 MET hours/week), or high (≥ 50 MET hours/week). Height and body weight were measured using a portable scale (YG-200DN; Yagami Inc., Nagoya, Japan), and the BMI was determined by dividing body weight in kilograms by the square of height in meters.

### Statistical analysis

The Wilcoxon rank-sum test and Fisher’s exact test were used to compare the continuous and categorical data, respectively, between participants with and without cancer.

Time-dependent Cox proportional hazards analysis was used as the main form of statistical analysis in this study, with cancer as an independent variable and overall FFs as a dependent variable. In addition, the following were used as independent variables in the statistical analysis: cancer treatment status, number of primary cancers, and cancer site. These models were used to estimate the hazard ratios (HRs) and 95% confidence intervals (CIs). The key time-dependent variables were cancer status and age, which changed during the follow-up period. For all participants, time zero was defined as the baseline survey and was followed up until the 10-year follow-up survey or the period from the baseline survey to the first FF among hip, vertebral compression, and distal radius fractures, whichever occurred first. However, participants who were diagnosed with cancer were transferred from the “without cancer” set to the “with cancer” set when cancer was diagnosed, thereby modifying their status from “without cancer” to “with cancer.” Transferees, deaths, and loss to follow-up were not assessed in this study because we only investigated individuals who participated in a 10-year follow-up survey. To examine the robustness of the association and the influence of potential confounders, the models were constructed in several steps, with sequential adjustment for demographic, anthropometric, and socioeconomic and lifestyle factors. We constructed the univariate models as model 1 and multivariate models, namely, model 2 with adjustment for age (years), sex (female or male), and menopausal status (premenopausal, perimenopausal, or postmenopausal); model 3 with further adjustment for BMI (kg/m^2^); and model 4 with further adjustment for highest educational attainment (below high school, high school, or college or trade school), number of comorbidities (zero, one, two, and three or more), history of hormone replacement therapy (yes or no), osteoporosis medication use (yes or no), corticosteroid use (yes or no), smoking status (non-smoker, past smoker, light smoker, moderate smoker, or heavy smoker), alcohol consumption (no, light, or moderate/heavy), physical activity (low, middle, or high), and total energy intake (kcal/day). For each year of follow-up from baseline, age was increased by one year. All other independent variables in the model, except for cancer status and age, were assumed to remain constant over the follow-up period, similar to previous studies^3–6^.

The post-hoc power analyses for the risk of FFs were estimated on the basis of the statistical estimates of time-dependent Cox proportional hazards models, such as HR, person-year, and number of events. An α level of 0.05 and ρ² level of 0.2^51^ were set.

To test the robustness of the associations observed in the main analyses, we conducted a sensitivity analysis by using Cox proportional hazards models; the follow-up was initiated from the time of cancer diagnosis, excluding the period from the baseline survey to cancer diagnosis in participants with newly diagnosed cancer during the follow-up period. Thus, these patients were not treated as cancer free but as having cancer at the start of follow-up.

All tests were two-sided. A *p* value < 0.050 was considered statistically significant. R statistical software version 4.5.0 (R Foundation) was used for the time-dependent Cox proportional hazards models and their post-hoc power analyses. JMP^®^ pro 17 (SAS Institute, Cary, North Carolina, USA) was used for all other statistical analyses.

## Results

### Characteristics of the study participants

Among the 10,330 participants included, 6148 (59.5%) were females (Table 1). The mean age was 56.4 years (median, 57.0 years). Among the 10,330 participants, 513 had a history of cancer at baseline and 9817 did not. During follow-up, 740 participants developed new cancers. Thus, 1253 cancer diagnoses were identified during the study period; the distribution of cancer diagnoses and follow-up duration by cancer type is shown in Supplementary Table 1.

**Table 1:** Baseline characteristics of participants according to cancer.

Female, *n* (%)	Overall (*n* = 10330)	With cancer (*n* = 513)	Without cancer (*n* = 9817)
	6148 (59.5)	310 (60.4)	5838 (59.5)
Menopausal status (female only)			
Premenopausal, *n* (%)	1505 (24.5)	30 (9.7)	1475 (25.3)
Perimenopausal, *n* (%)	429 (7.0)	13 (4.2)	416 (7.1)
Postmenopausal, *n* (%)	4214 (68.5)	267 (86.1)	3947 (67.6)
Age, years	57.0 (50.0, 63.0)	56.0 (49.0, 62.0)	57.0 (50.0, 63.0)
40–49 years, *n* (%)	2467 (23.9)	143 (27.9)	2324 (23.7)
50–59 years, *n* (%)	3830 (37.1)	197 (38.4)	3633 (37.0)
60–69 years, *n* (%)	4033 (39.0)	173 (33.7)	3860 (39.3)
Body mass index, kg/m^2^	22.6 (20.7, 24.6)	22.5 (20.5, 24.7)	22.6 (20.7, 24.6)
<18.5 kg/m^2^, *n* (%)	529 (5.1)	30 (5.9)	499 (5.1)
18.5–24.9 kg/m^2^, *n* (%)	7589 (73.5)	366 (71.4)	7223 (73.6)
≥25.0 kg/m^2^, *n* (%)	2212 (21.4)	117 (22.8)	2095 (21.3)
Highest educational attainment			
< High school, *n* (%)	581 (5.6)	38 (7.4)	543 (5.5)
High school, *n* (%)	5189 (50.2)	263 (51.3)	4926 (50.2)
College or trade school, *n* (%)	4560 (44.1)	212 (41.3)	4348 (44.3)
Number of comorbidities			
0, *n* (%)	5144 (49.8)	256 (49.9)	4888 (49.8)
1, *n* (%)	3295 (31.9)	152 (29.6)	3143 (32.0)
2, *n* (%)	1452 (14.1)	79 (15.4)	1373 (14.0)
≥3, *n* (%)	439 (4.3)	26 (5.1)	413 (4.2)
History of hormone replacement therapy (female only), *n* (%)	704 (11.5)	80 (25.8)	624 (10.7)
Osteoporosis medication use, *n* (%)	130 (1.3)	9 (1.8)	121 (1.2)
Corticosteroid use, *n* (%)	120 (1.2)	12 (2.3)	108 (1.1)
Smoking status			
Non-smoker, *n* (%)	7464 (72.3)	388 (75.6)	7076 (72.1)
Past smoker, *n* (%)	1295 (12.5)	48 (9.4)	1247 (12.7)
Light smoker (≤ 9 cigarettes/day), *n* (%)	113 (1.1)	3 (0.6)	110 (1.1)
Moderate smoker (10–19 cigarettes/day), *n* (%)	365 (3.5)	17 (3.3)	348 (3.5)
Heavy smoker (≥ 20 cigarettes/day), *n* (%)	1093 (10.6)	57 (11.1)	1036 (10.6)
Alcohol consumption, unit/day	0.1 (0, 0.8)	0 (0, 0.6)	0.1 (0, 0.8)
None (0 unit/day), *n* (%)	4717 (45.7)	264 (51.5)	4453 (45.4)
Light (1–2 unit/day), *n* (%)	5058 (49.0)	226 (44.1)	4832 (49.2)
Moderate/Heavy (≥ 3 unit/day), *n* (%)	555 (5.4)	23 (4.5)	532 (5.4)
Physical activity			
Low, *n* (%)	5631 (54.5)	276 (53.8)	5355 (54.6)
Moderate, *n* (%)	4154 (40.2)	212 (41.3)	3942 (40.2)
High, *n* (%)	545 (5.3)	25 (4.9)	520 (5.3)
Total energy intake (kcal/day)	1636.1 (1465.3, 1877.2)	1626.5 (1436.5, 1865.6)	1636.5 (1466.2, 1877.7)

Continuous values are shown as medians (interquartile range).

Categorical values are shown as numbers (percentage).

There were no missing data.

Among the 605 participants who experienced fractures due to falls from standing positions during follow-up, 386 had FFs (hip, vertebral compression, or distal radius fractures). Among the 386 participants with FFs, 37 had a history of cancer at the baseline survey, 332 had no history of cancer at the baseline survey and did not develop cancer during follow-up, and 17 had no history of cancer at the baseline survey but developed cancer during follow-up (3 and 14 participants had FFs before and after cancer onset, respectively; these fractures were analyzed with consideration to the cancer status).

Median follow-up period for the cohort was 10.0 years (interquartile range 9.9–10.0). Among participants who experienced FFs, the median time from baseline to the first fracture was 7.0 years (interquartile range, 5.0–8.0) overall, 7.0 years (interquartile range, 5.0–9.0) in those with cancer at baseline, and 7.0 years (interquartile range, 4.8–8.0) in those without cancer at baseline. The overall incidence rates of overall FFs in participants with and without cancer were 6.4 and 3.5 per 1000 person-years, respectively (Table 2).

**Table 2:** Time-dependent Cox proportional hazards models of number of years from baseline survey to overall FFs.

				HR (95% CI)			
	Event	Person-year	IR (per 1000)	Model 1	Model 2	Model 3	Model 4
*Entire participants*							
No cancer	335	94,382	3.5	1.00 (Reference)	1.00 (Reference)	1.00 (Reference)	1.00 (Reference)
Cancer	51	8010	6.4	**1.61 (1.19–2.17)**	**1.40 (1.03–1.89)**	**1.42 (1.05–1.91)**	**1.40 (1.03–1.89)**
*Female participants*							
No cancer	252	55,970	4.5	1.00 (Reference)	1.00 (Reference)	1.00 (Reference)	1.00 (Reference)
Cancer	33	4779	6.9	1.39 (0.97–2.01)	1.19 (0.83–1.72)	1.20 (0.84–1.74)	1.18 (0.82–1.71)
*Male participants*							
No cancer	83	38,412	2.2	1.00 (Reference)	1.00 (Reference)	1.00 (Reference)	1.00 (Reference)
Cancer	18	3231	5.6	**2.25 (1.33–3.80)**	**2.07 (1.22–3.51)**	**2.11 (1.24–3.57)**	**2.03 (1.20–3.44)**
*Cancer treatment status*							
No cancer	335	94,382	3.5	1.00 (Reference)	1.00 (Reference)	1.00 (Reference)	1.00 (Reference)
Previous cancer	23	4366	5.3	1.43 (0.93–2.18)	1.13 (0.73–1.73)	1.15 (0.75–1.77)	1.14 (0.74–1.76)
Current cancer	28	3644	7.7	**1.80 (1.22–2.67)**	**1.74 (1.17–2.58)**	**1.74 (1.18–2.59)**	**1.71 (1.15–2.55)**
*Number of primary cancers*							
No cancer	335	94,382	3.5	1.00 (Reference)	1.00 (Reference)	1.00 (Reference)	1.00 (Reference)
Single primary cancer	46	7261	6.3	1.55 (0.64–3.74)	1.14 (0.47–2.76)	1.16 (0.48–2.83)	1.18 (0.49–2.87)
Multiple primary cancer	5	749	6.7	**1.62 (1.18–2.21)**	**1.43 (1.05–1.96)**	**1.45 (1.06–1.98)**	**1.42 (1.04–1.95)**
*Cancer site*							
No cancer	335	94,382	3.5	1.00 (Reference)	1.00 (Reference)	1.00 (Reference)	1.00 (Reference)
Stomach cancer	11	1545	7.1	**1.89 (1.04–3.45)**	**1.85 (1.01–3.38)**	**1.84 (1.01–3.36)**	**1.84 (1.01–3.37)**
Colon and rectum cancer	5	1284	3.9	1.00 (0.42–2.41)	0.90 (0.37–2.18)	0.91 (0.38–2.21)	0.90 (0.47–2.20)
Thyroid cancer	1	204	4.9	1.36 (0.19–9.67)	1.29 (0.18–9.16)	1.31 (0.18–9.37)	1.40 (0.20–10.0)
Bladder cancer	2	247	8.1	2.08 (0.52–8.34)	1.87 (0.46–7.51)	1.88 (0.47–7.57)	1.97 (0.49–7.93)
Kidney cancer	3	155	19.4	**4.66 (1.49–14.53)**	**3.52 (1.13–11.01)**	**3.57 (1.14–11.14)**	**3.74 (1.20–11.70)**
Hematologic cancer	3	124	24.2	**7.04 (2.26–21.93)**	**8.87 (2.84–27.67)**	**8.05 (2.58–25.15)**	**7.70 (2.45–24.16)**
Breast cancer	9	1486	6.1	1.41 (0.70–2.85)	1.09 (0.54–2.20)	1.10 (0.54–2.22)	1.04 (0.51–2.12)
Gynecological cancer	6	1133	5.3	1.46 (0.65–3.27)	1.23 (0.55–2.77)	1.26 (0.56–2.83)	1.18 (0.52–2.66)
Prostate cancer	3	552	5.4	1.26 (0.40–3.92)	0.89 (0.28–2.78)	0.93 (0.30–2.92)	0.95 (0.30–2.97)
The other cancers	8	1280	6.3	1.50 (0.74–3.02)	1.40 (0.69–2.82)	1.41 (0.70–2.86)	1.43 (0.71–2.89)

*FF* fragility fracture, *HR* hazard ratio, *CI* confidence interval, *IR* incidence rate. Bold values indicate statistical significance.

Model 1: unadjusted.

Model 2: adjusted for age, sex, and menopausal status.

Model 3: model 2 plus body mass index.

Model 4: model 3 plus highest educational attainment, number of comorbidities, history of hormone replacement therapy, osteoporosis medication use, corticosteroid use, smoking status, alcohol consumption, physical activity, and total energy intake.

During follow-up, 10 participants (0.1%) experienced multiple fractures; participants with cancer experienced significantly more multiple fractures than those without cancer (0.6% [*n* = 3] vs. 0.1% [*n* = 7], *p* = 0.011). Among participants with FFs, three cancer survivors experienced FFs at more than one site.

Table 1 shows that cancer survivors had significantly higher corticosteroid use (2.3% vs. 1.1%, *p* = 0.018) and lower alcohol consumption (0 unit/day vs. 0.1 unit/day, *p* = 0.009) than participants without cancer. Female cancer survivors were significantly more likely to be postmenopausal (86.1% vs. 67.6%, *p* < 0.001) and to have a history of hormone replacement therapy (25.8% vs. 10.7%, *p* < 0.001) than female participants without cancer.

### Main analyses

The direction and magnitude of the associations were generally consistent across Models 1–4, indicating robustness of the findings. In model 4, which was adjusted for all potential confounders (Table 2), cancer survivors had a statistically significant higher risk of overall FFs in all participants (HR, 1.40; 95% CI, 1.03–1.89) and male participants (HR, 2.03; 95% CI, 1.20–3.44) than participants without cancer, whereas there was no significant difference in female participants (HR, 1.18; 95% CI, 0.82–1.71). This risk was further elevated among participants with active cancer (HR, 1.71; 95% CI, 1.15–2.55) and multiple primary cancers (HR, 1.42; 95% CI, 1.04–1.95). The risks of overall FFs were significantly higher in cancer survivors for stomach (HR, 1.84; 95% CI, 1.01–3.37), kidney (HR, 3.74; 95% CI, 1.20–11.70), and hematologic cancers (HR, 7.70; 95% CI, 2.45–24.16) than in participants without cancer; however, no significant difference was observed for the other cancers.

Table 3 shows the HRs for site-specific FFs in cancer survivors. Cancer survivors had a statistically significant higher risk of vertebral compression fracture (HR, 1.56; 95% CI, 1.01–2.43) and marginal significant higher risk of distal radius fracture (HR, 1.43; 95% CI, 0.93–2.19; *p* = 0.099) than participants without cancer, whereas no significant difference was observed for hip fracture (HR, 0.99; 95% CI, 0.35–2.78).

**Table 3:** Time-dependent Cox proportional hazards models of number of years from baseline survey to site-specific FFs (i.e., hip, vertebral compression, and distal radius fractures).

				HR (95% CI)			
	Event	Person-year	IR (per 1000)	Model 1	Model 2	Model 3	Model 4
*Hip fracture*							
No cancer	41	95,427	0.4	1.00 (Reference)	1.00 (Reference)	1.00 (Reference)	1.00 (Reference)
Cancer	4	8167	0.5	1.03 (0.37–2.89)	0.90 (0.32–2.52)	0.93 (0.33–2.60)	0.99 (0.35–2.78)
*Vertebral* compression *fracture*							
No cancer	137	95,107	1.4	1.00 (Reference)	1.00 (Reference)	1.00 (Reference)	1.00 (Reference)
Cancer	25	8110	3.1	**1.84 (1.19–2.85)**	**1.56 (1.01–2.43)**	**1.58 (1.02–2.46)**	**1.56 (1.01–2.43)**
*Distal radius fracture*							
No cancer	165	94,950	1.7	1.00 (Reference)	1.00 (Reference)	1.00 (Reference)	1.00 (Reference)
Cancer	25	8082	3.1	**1.67 (1.09–2.55)**	1.47 (0.96–2.24)	1.49 (0.97–2.27)	1.43 (0.93–2.19)

*FF* fragility fracture, *HR* hazard ratio, *CI* confidence interval, *IR* incidence rate. Bold values indicate statistical significance.

Model 1: unadjusted.

Model 2: adjusted for age, sex, and menopausal status.

Model 3: model 2 plus body mass index.

Model 4: model 3 plus highest educational attainment, number of comorbidities, history of hormone replacement therapy, osteoporosis medication use, corticosteroid use, smoking status, alcohol consumption, physical activity, and total energy intake.

### Post-hoc power analyses

The estimated statistical power was 37.4% for overall FF risk, 9.9% for females, 47.8% for males, 2.6% for hip fractures, 27.5% for vertebral compression fractures, and 21.9% for distal radius fractures. These statistical powers suggested that the ability to detect a statistically significant association between cancer diagnosis and FF risk was limited, particularly in subgroups with a small number of events.

### Sensitivity analyses

In model 4, which was adjusted for all potential confounders (Table 4), cancer survivors had a statistically significant higher risk of overall FFs in all participants (HR, 2.04; 95% CI, 1.53–2.70), female participants (HR, 1.72; 95% CI, 1.22–2.43), and male participants (HR, 3.02; 95% CI, 1.84–4.95) than participants without cancer. The risk of overall FFs was significantly higher in participants with previous cancer diagnosis (HR 1.63, 95% CI 1.10–2.41) than in those without cancer, and substantially greater in those with active cancer (HR 2.65, 95% CI 1.81–3.89). An increased risk of overall FFs was observed in participants with a single primary cancer (HR 2.02, 95% CI 1.50–2.71) compared with those without cancer, and the risk was even higher among those with multiple primary cancers (HR 2.22, 95% CI 1.00–5.02). The risks of overall FFs were significantly elevated in survivors of stomach cancer (HR 2.40, 95% CI 1.35–4.29), kidney cancer (HR 5.48, 95% CI 1.75–17.16), and hematologic cancers (HR 8.10, 95% CI 2.58–25.50) compared with those without cancer. However, no significant differences were detected for the other cancers. All HRs were higher in the sensitivity analyses than in the main analyses.

**Table 4:** Sensitivity analyses excluding the period preceding cancer diagnosis as well as Cox proportional hazards model analysis of number of years from baseline survey or cancer diagnosis to overall FFs.

				HR (95% CI)			
	Event	Person-year	IR (per 1000)	Model 1	Model 2	Model 3	Model 4
*Entire participants*							
No cancer	332	90,008	3.7	1.00 (Reference)	1.00 (Reference)	1.00 (Reference)	1.00 (Reference)
Cancer	51	8018	6.4	**2.27 (1.72–3.00)**	**2.04 (1.54–2.70)**	**2.06 (1.56–2.72)**	**2.04 (1.53–2.70)**
*Female participants*							
No cancer	251	53,156	4.7	1.00 (Reference)	1.00 (Reference)	1.00 (Reference)	1.00 (Reference)
Cancer	33	4786	6.9	**1.98 (1.41–2.77)**	**1.73 (1.23–2.44)**	**1.75 (1.24–2.45)**	**1.72 (1.22–2.43)**
*Male participants*							
No cancer	81	36,852	2.2	1.00 (Reference)	1.00 (Reference)	1.00 (Reference)	1.00 (Reference)
Cancer	18	3232	5.6	**3.15 (1.93–5.15)**	**3.06 (1.87–5.01)**	**3.10 (1.89–5.08)**	**3.02 (1.84–4.95)**
*Cancer treatment status*							
No cancer	332	90,008	3.7	1.00 (Reference)	1.00 (Reference)	1.00 (Reference)	1.00 (Reference)
Previous cancer	23	4366	5.3	**1.74 (1.19–2.56)**	**1.66 (1.13–2.45)**	**1.67 (1.13–2.45)**	**1.63 (1.10–2.41)**
Current cancer	28	3652	7.7	**3.16 (2.17–4.60)**	**2.60 (1.78–3.80)**	**2.64 (1.81–3.87)**	**2.65 (1.81–3.89)**
*Number of primary cancers*							
No cancer	332	90,008	3.7	1.00 (Reference)	1.00 (Reference)	1.00 (Reference)	1.00 (Reference)
Single primary cancer	46	7261	6.3	**2.23 (1.66–2.98)**	**2.03 (1.52–2.72)**	**2.05 (1.53–2.74)**	**2.02 (1.50–2.71)**
Multiple primary cancer	5	757	6.6	**2.78 (1.24–6.24)**	2.14 (0.95–4.81)	2.16 (0.96–4.87)	**2.22 (1.00–5.02)**
*Cancer site*							
No cancer	332	90,008	3.7	1.00 (Reference)	1.00 (Reference)	1.00 (Reference)	1.00 (Reference)
Stomach cancer	11	1545	7.1	**2.36 (1.32–4.19)**	**2.44 (1.37–4.35)**	**2.41 (1.36–4.31)**	**2.40 (1.35–4.29)**
Colon and rectum cancer	5	1284	3.9	1.20 (0.50–2.90)	1.16 (0.48–2.81)	1.17 (0.48–2.84)	1.15 (0.48–2.80)
Thyroid cancer	1	204	4.9	1.39 (0.19–9.88)	1.39 (0.19–9.89)	1.40 (0.20–10.01)	1.48 (0.21–10.56)
Bladder cancer	2	247	8.1	2.57 (0.64–10.32)	2.68 (0.66–10.80)	2.67 (0.66–10.77)	2.80 (0.69–11.29)
Kidney cancer	3	155	19.4	**6.04 (1.94–18.82)**	**5.12 (1.64–15.97)**	**5.13 (1.64–16.02)**	**5.48 (1.75–17.16)**
Hematologic cancer	3	124	24.2	**6.96 (2.23–21.69)**	**9.06 (2.90–28.28)**	**8.39 (2.69–26.24)**	**8.10 (2.58–25.50)**
Breast cancer	9	1486	6.1	**2.45 (1.38–4.37)**	**1.83 (1.03–3.26)**	**1.84 (1.03–3.28)**	1.77 (0.98–3.21)
Gynecological cancer	6	1140	5.3	1.77 (0.84–3.75)	1.42 (0.67–3.00)	1.45 (0.68–3.08)	1.39 (0.65–2.95)
Prostate cancer	3	552	5.4	1.96 (0.63–6.12)	1.44 (0.46–4.50)	1.49 (0.47–4.67)	1.53 (0.49–4.80)
The other cancers	8	1281	6.2	**2.81 (1.54–5.13)**	**2.98 (1.62–5.45)**	**2.99 (1.63–5.47)**	**3.03 (1.65–5.55)**

*FF* fragility fracture, *HR* hazard ratio, *CI* confidence interval, *IR* incidence rate. Bold values indicate statistical significance.

Model 1: unadjusted.

Model 2: adjusted for age, sex, and menopausal status.

Model 3: model 2 plus body mass index.

Model 4: model 3 plus highest educational attainment, number of comorbidities, history of hormone replacement therapy, osteoporosis medication use, corticosteroid use, smoking status, alcohol consumption, physical activity, and total energy intake.

Table 5 shows that cancer survivors had a statistically significant higher risk of vertebral compression fracture (HR, 2.19; 95% CI, 1.42–3.39) and distal radius fracture (HR, 1.65; 95% CI, 1.07–2.53) than participants without cancer, and no significant difference was observed for hip fracture (HR, 1.27; 95% CI, 0.45–3.59).

**Table 5:** Sensitivity analyses excluding the period preceding cancer diagnosis as well as Cox proportional hazards model analysis of number of years from baseline survey or cancer diagnosis to site-specific FFs (i.e., hip, vertebral compression, and distal radius fractures).

				HR (95% CI)			
	Event	Person-year	IR (per 1000)	Model 1	Model 2	Model 3	Model 4
*Hip fracture*							
No cancer	41	91,050	0.5	1.00 (Reference)	1.00 (Reference)	1.00 (Reference)	1.00 (Reference)
Cancer	4	8167	0.5	1.26 (0.45–3.53)	1.15 (0.41–3.25)	1.18 (0.42–3.30)	1.27 (0.45–3.59)
*Vertebral compression fracture*							
No cancer	136	90,731	1.5	1.00 (Reference)	1.00 (Reference)	1.00 (Reference)	1.00 (Reference)
Cancer	25	8111	3.1	**2.37 (1.54–3.63)**	**2.17 (1.41–3.34)**	**2.19 (1.42–3.37)**	**2.19 (1.42–3.39)**
*Distal radius fracture*							
No cancer	163	90,576	1.8	1.00 (Reference)	1.00 (Reference)	1.00 (Reference)	1.00 (Reference)
Cancer	25	8090	3.1	**1.93 (1.27–2.95)**	**1.70 (1.11–2.59)**	**1.71 (1.12–2.61)**	**1.65 (1.07–2.53)**

*FF* fragility fracture, *HR* hazard ratio, *CI* confidence interval, *IR* incidence rate. Bold values indicate statistical significance.

Model 1: unadjusted.

Model 2: adjusted for age, sex, and menopausal status.

Model 3: model 2 plus body mass index.

Model 4: model 3 plus highest educational attainment, number of comorbidities, history of hormone replacement therapy, osteoporosis medication use, corticosteroid use, smoking status, alcohol consumption, physical activity, and total energy intake.

In the sensitivity analysis, the direction and magnitude of the association between cancer and overall FFs remained consistent with those observed in the main analysis.

## Discussion

This study, which utilized time-dependent Cox proportional hazards models, provides compelling evidence that cancer survivors have a significantly higher risk of developing FFs than individuals without a history of cancer. This elevated risk was particularly pronounced among those with active cancer, multiple primary cancers, and specific cancer types, notably stomach, kidney, and hematologic cancers. Furthermore, our analysis revealed distinct patterns in fracture localization, with significantly higher risks observed for vertebral compression and distal radius fractures in cancer survivors.

Our main analysis revealed that cancer survivors had lower HRs for FFs than in previous findings^3^. This outcome is attributable to our inclusive approach, wherein the entire follow-up period was considered, and the pre-diagnosis time for cancer survivors was appropriately categorized as non-cancer person-time. This methodology inherently yields a more conservative risk estimate. By contrast, both our sensitivity analysis and that of a previous study^3^ excluded the pre-diagnosis period, i.e., fracture events were calculated solely from the point of cancer diagnosis. This exclusion usually leads to the overestimation of fracture risk because it disregards events that occur during the latent phase of cancer development. Consequently, our main analysis provides a more comprehensive and reliable assessment of FF risk in this population.

The primary findings of this study can be explained by three potential biological mechanisms: cancer treatments, the cancer itself, and associated chronic psychological stress. First, cancer treatments, such as radiation, hormone deprivation therapy, and surgery, can affect bone remodeling^2,12,40^ across various cancer types. For example, radiation therapy may cause localized bone damage and alter bone metabolism by reducing osteoblast activity and increasing osteoclast activity, as observed in pelvic fractures after colorectal cancer treatments^11^. Hormonal therapies decrease sex hormone levels, thereby accelerating bone turnover and reducing bone mineral density^16,17,30^. Gastrointestinal surgeries (e.g., gastrectomy) impair calcium and vitamin D absorption, thus limiting bone formation^7–10^. Second, cancer itself can disrupt normal bone remodeling through various biological pathways. For instance, hematologic malignancies also directly impair bone remodeling by stimulating osteoclastic activity and suppressing osteoblastic function^27–29^. Third, chronic psychological stress due to cancer and its treatment negatively affects bone remodeling through physiological changes, such as increased glucocorticoids, increased prolactin, increased leptin, increased parathyroid hormone levels, reduced gonadal hormones, low-grade inflammation, and sympathetic nervous system hyperactivation^52^. Collectively, the biological effect of cancer and its treatments on bone remodeling may increase the risk of developing FFs.

Interestingly, we found that Japanese individuals with active cancer had a significantly higher risk of overall FFs than those with previous cancer diagnosis. As discussed above, the deleterious effects of cancer itself and/or cancer therapy may gradually disappear over time. Consistently, Rees-Punia et al.^3^ and Buzasi et al.^4^ reported that the increased risk of fracture persisted beyond five years from diagnosis in several cancers (namely, stomach, breast, gynecological, colon and rectum, bladder, prostate, kidney, and hematologic cancers), but the magnitude of the association tended to decrease over time after cancer diagnosis.

One novel finding in this study is that participants with multiple primary cancers had a significantly higher risk of overall FFs than those with a single primary cancer. However, the underlying mechanisms were not elucidated in this study. Given that all cancer diagnoses at baseline were self-reported, metastatic cancers, which often involve fracture-prone sites^3,41^, might have been misclassified as multiple primary cancers. This potential misclassification could partially explain the elevated FF risk in this group because individuals with unrecognized metastases would inherently be at a higher risk of fracture. The increased risk of FF associated with multiple primary cancers is a clinically important finding. Future studies should investigate the cumulative effects and interactions between different cancers and their associated treatments.

Regarding cancer sites in the Japanese population, we observed significantly higher risks of overall FF in survivors of stomach, kidney, and hematological cancers than in those without cancer; this finding is consistent with those of previous studies involving other ethnicities^15,18,38^. In Japan, where the incidence of gastric cancer remains relatively high owing to nationwide screening programs and dietary factors, the higher number of gastrectomies performed as standard treatment has contributed to improved survival outcomes^53^; however, this surgical approach could also increase the risk of post-gastrectomy syndrome, including osteoporosis. Immune checkpoint inhibitors are an important treatment option for kidney cancer, but they may affect bone remodeling and potentially increase the risk of vertebral compression fractures^26^. Hematologic cancer disrupts bone remodeling and causes bone loss and osteoporosis, and its treatments and impairment of peak bone mass lead to increased fracture risk in survivors^27–29^.

No significant increase in overall FF risk was observed for colon, rectal, thyroid, bladder, breast, gynecological, and prostate cancers in this study even though previous studies have reported significantly elevated risks for these cancer types^11–18,24,25,30–37^. A primary contributing factor to this discrepancy might be the insufficient statistical power in the present study because of the relatively small number of patients with these specific cancer types and the limited number of fracture events observed. Furthermore, although a study^4^ examining fracture risk across 20 cancer types highlighted concerns regarding multiple testing, the current study rigorously addressed this issue by assessing the FF risk in 10 predefined cancer types. Even after applying a conservative Bonferroni correction (adjusted significance level of *p* < 0.005 based on a nominal *p* < 0.05), only hematologic cancer remained statistically significant, thus underscoring its particularly high FF risk. Despite the limitation caused by the potentially insufficient sample sizes for certain cancer types, the present results could facilitate the prioritization of specific cancer sites at a demonstrably high risk of FFs.

Cancer survivors had a marginally significant higher risk of distal radius fractures. By contrast, Rees-Punia et al.^3^ reported that cancer survivors did not have a significantly higher risk of distal radius fractures but had a significantly higher risk of hip fractures. This discrepancy between our results and those of Rees-Punia et al.^3^ may be due to the differences in the ages of the study subjects. First, the current study included a relatively younger population, whereas Rees-Punia et al.^3^ analyzed a large elderly population that was physically inactive and more susceptible to falls and hip fractures^39^. We speculate that distal radius fractures are more likely to occur in active and relatively healthy adults who have functional mobility and can participate in a wide range of activities.

### Strengths and limitations of the study

This study provides valuable longitudinal epidemiologic evidence on the overall risk of FFs among cancer survivors in the general Asian population. A careful consideration of time-dependent variables and adjustment for multiple detailed covariates enabled us to accurately identify the increased risk of overall FFs in Japanese cancer survivors. Another strength and novel aspect of this study is the secondary analyses that evaluated fracture risk by treatment status, number of primary cancers, and cancer site.

However, this study has a few limitations. First, reporting bias might be present because the history of cancer at the baseline survey was self-reported and was not verified via medical records. In addition, detailed information on cancer therapies and staging was not available. Thus, FFs could not be distinguished from pathologic fractures due to bone metastases. Although self-reported cancer information before the baseline survey was not validated in this cohort, previous studies have shown that self-reported cancer diagnoses have acceptable validity (positive predictive value: 61.2%–97.5%)^54^, thus supporting their utility for estimating fracture risk in cancer survivors. Second, all covariates included in the time-dependent Cox proportional hazards models, except BMI, were self-reported. Drugs were counted only when the information was provided by participants, thus leading to oversights or misclassifications. For instance, no information is available regarding injectable medications for outpatient treatments or the duration of treatment, such as for osteoporosis. Third, the statistical power, particularly in the secondary analyses that used cancer treatment status, number of primary cancers, and cancer site as independent variables and the specific FF site as a dependent variable, was limited because of the small number of fracture events in several subgroups; therefore, these findings should be interpreted cautiously and considered exploratory. Further stratified analyses by lifestyle factors (e.g., smoking, body mass index, and diabetes) and age at cancer diagnosis were not feasible because of the limited number of fracture events in the cancer subgroup. Fourth, because the analyses were restricted to participants who completed the 10-year follow-up survey, transferees, deaths, and loss to follow-up during follow-up were not included, which may have introduced selection and survival bias. In particular, participants who developed cancer and subsequently experienced FFs but died before completing follow-up may have been excluded, potentially leading to an underestimation of the association between cancer and FFs. Therefore, competing risk analyses could not be performed. Fifth, some cancers, such as breast and prostate cancer, have treatment pathways that affect bone metabolism through hormonal deprivation therapy, and bone health is often considered as part of their comprehensive care; however, this aspect was not evaluated in the present study.

In conclusion, by using time-dependent Cox proportional hazards models, we investigated the true epidemiological risk of overall FFs in Japanese cancer survivors and found that they had a significantly higher risk of overall FFs than those without cancer. These findings highlight the importance of FF prevention strategies in cancer survivorship care in aging Asian populations.

## Supplementary Information

Below is the link to the electronic supplementary material.

Supplementary Material 1

## Data Availability

The datasets used during the current study are not publicly available because of patient confidentiality but are available from the corresponding author on reasonable request.

## References

[1] Kawano, H., Hirahata, M. & Imanishi, J. Locomotive syndrome in cancer patients: A new role of orthopaedic surgeons as a part of comprehensive cancer care. *Int. J. Clin. Oncol.* **27**, 1233–1237 (2022). 35690700 10.1007/s10147-022-02194-w PMC9309135

[2] Ye, C. & Leslie, W. D. Fracture risk and assessment in adults with cancer. *Osteoporos. Int.* **34**, 449–466 (2023). 36512057 10.1007/s00198-022-06631-4

[3] Rees-Punia, E. et al. Fracture risk among older cancer survivors compared with older adults without a history of cancer. *J. M Oncol.* **9**, 79–87 (2023). 10.1001/jamaoncol.2022.5153 PMC9634602 36326746

[4] Buzasi, E. et al. Risk of fractures in half a million survivors of 20 cancers: A population-based matched cohort study using linked English electronic health records. *Lancet Healthy Longev.* **5**, e194–e203 (2024). 38335985 10.1016/S2666-7568(23)00285-4 PMC10904352

[5] Gong, I. Y., Chan, K. K. W., Lipscombe, L. L., Cheung, M. C. & Mozessohn, L. Fracture risk among patients with cancer compared to individuals without cancer: A population-based study. *Br. J. Cancer*. **129**, 665–671 (2023). 37422530 10.1038/s41416-023-02353-4 PMC10421906

[6] Vestergaard, P., Rejnmark, L. & Mosekilde, L. Fracture risk in patients with different types of cancer. *Acta Oncol.* **48**, 105–115 (2009). 18607871 10.1080/02841860802167490

[7] Seo, G. H., Kang, H. Y. & Choe, E. K. Osteoporosis and fracture after gastrectomy for stomach cancer: A nationwide claims study. *Med. (Baltim).* **97**, e0532 (2018). 10.1097/MD.0000000000010532 PMC5944502 29703028

[8] Oh, H. et al. The risk of osteoporotic fracture in gastric cancer survivors: total gastrectomy versus subtotal gastrectomy versus endoscopic treatment. *Gastric Cancer*. **26**, 814–822 (2023). 37209225 10.1007/s10120-023-01397-y

[9] Iki, M. et al. Increased risk of osteoporotic fracture in community-dwelling elderly men 20 or more years after gastrectomy: The Fujiwara-kyo Osteoporosis Risk in Men (FORMEN) Cohort Study. *Bone* **127**, 250–259 (2019). 31254731 10.1016/j.bone.2019.06.014

[10] Shin, D. W. et al. Increased risk of osteoporotic fracture in postgastrectomy gastric cancer survivors compared with matched controls: A nationwide cohort study in Korea. *Am. J. Gastroenterol.* **114**, 1735–1743 (2019). 31658122 10.14309/ajg.0000000000000436

[11] Kang, Y. M., Chao, T. F., Wang, T. H. & Hu, Y. W. Increased risk of pelvic fracture after radiotherapy in rectal cancer survivors: A propensity matched study. *Cancer Med.* **8**, 3639–3647 (2019). 31104362 10.1002/cam4.2030 PMC6639197

[12] Ku, E. J. et al. Risk of osteoporotic fractures among patients with thyroid cancer: A nationwide population-based cohort study. *Endocrinol. Metab. (Seoul)*. **40**, 225–235 (2025). 39814032 10.3803/EnM.2024.2101 PMC12061740

[13] Tsai, C. H. et al. Fracture in Asian women with breast cancer occurs at younger age. *PLoS One*. **8**, e75109 (2013). 24069386 10.1371/journal.pone.0075109 PMC3771894

[14] Kawamura, C. et al. Non-cancer risks among female breast cancer survivors: a matched cohort study in Japan. *Lancet Reg. Health West. Pac.* **56**, 101519 (2025). 40206327 10.1016/j.lanwpc.2025.101519 PMC11979481

[15] Kim, D. K. et al. Androgen-deprivation therapy and the risk of newly developed fractures in patients with prostate cancer: a nationwide cohort study in Korea. *Sci. Rep.* **11**, 10057 (2021). 33980958 10.1038/s41598-021-89589-3 PMC8115250

[16] Matsushima, H. et al. Androgen deprivation therapy-related fracture risk in prostate cancer: An insurance claims database study in Japan. *J. Bone Min. Metab.* **42**, 223–232 (2024). 10.1007/s00774-024-01497-4 PMC10982088 38493435

[17] Taguchi, T. et al. Osteoporotic fracture risk in women with breast cancer treated with aromatase inhibitors: A health insurance claims database study in Japan. *Expert Opin. Pharmacother*. **25**, 325–334 (2024). 38588537 10.1080/14656566.2024.2340712

[18] Shin, H. B. et al. Risk of fracture incidence in prostate cancer survivors: A nationwide cohort study in South Korea. *Arch. Osteoporos.* **15**, 110 (2020). 32700143 10.1007/s11657-020-00785-6

[19] Choi, H. G. et al. Analyses of the association between breast cancer and osteoporosis/fracture history: A cross-sectional study using KoGES HEXA data. *Arch. Osteoporos.* **16**, 98 (2021). 34148148 10.1007/s11657-021-00947-0

[20] Lin, C. Y. et al. The impact of novel hormonal agents on fracture risk in prostate cancer patients: A nationwide population-based cohort study. *Sci. Rep.* **14**, 26696 (2024). 39496647 10.1038/s41598-024-73598-z PMC11535545

[21] Arbel, R. et al. Nirmatrelvir use and severe Covid-19 outcomes during the Omicron Surge. *N Engl. J. Med.* **387**, 790–798 (2022). 36001529 10.1056/NEJMoa2204919 PMC9454652

[22] Lévesque, L. E., Hanley, J. A., Kezouh, A. & Suissa, S. Problem of immortal time bias in cohort studies: example using statins for preventing progression of diabetes. *B M J.* **340**, b5087 (2010). 10.1136/bmj.b5087 20228141

[23] Pawloski, P. A. et al. Fracture risk in older, long-term survivors of early-stage breast cancer. *J. Am. Geriatr. Soc.* **61**, 888–895 (2013). 23647433 10.1111/jgs.12269 PMC3686911

[24] Bassett, J. H. & Williams, G. R. Role of thyroid hormones in skeletal development and bone maintenance. *Endocr. Rev.* **37**, 135–187 (2016). 26862888 10.1210/er.2015-1106 PMC4823381

[25] Gupta, A. et al. Risk of fracture after radical cystectomy and urinary diversion for bladder cancer. *J. Clin. Oncol.* **32**, 3291–3298 (2014). 25185104 10.1200/JCO.2013.54.3173 PMC4178526

[26] Filippini, D. M. et al. Bone fracture as a novel immune-related adverse event with immune checkpoint inhibitors: Case series and large-scale pharmacovigilance analysis. *Int. J. Cancer*. **149**, 675–683 (2021). 33844854 10.1002/ijc.33592 PMC8251715

[27] Thorsteinsdottir, S. et al. Fractures and survival in multiple myeloma: Results from a population-based study. *Haematologica* **105**, 1067–1073 (2020). 31792034 10.3324/haematol.2019.230011 PMC7109735

[28] Johansson, P. et al. Increased risk of hip fracture in patients with lymphoma, a Swedish population study of 37,236 lymphoma patients. *Calcif Tissue Int.* **106**, 591–598 (2020). 32170330 10.1007/s00223-020-00674-7

[29] Haddy, T. B., Mosher, R. B. & Reaman, G. H. Osteoporosis in survivors of acute lymphoblastic leukemia. *Oncologist* **6**, 278–285 (2001). 11423675 10.1634/theoncologist.6-3-278

[30] Kwan, M. L. et al. A prospective study of lifestyle factors and bone health in breast cancer patients who received aromatase inhibitors in an integrated healthcare setting. *J. Cancer Surviv*. **17**, 139–149 (2023). 33565036 10.1007/s11764-021-00993-0 PMC8349930

[31] Stumpf, U., Kostev, K., Kyvernitakis, J., Böcker, W. & Hadji, P. Incidence of fractures in young women with breast cancer - A retrospective cohort study. *J. Bone Oncol.* **18**, 100254 (2019). 31440445 10.1016/j.jbo.2019.100254 PMC6698802

[32] Fraenkel, M. et al. Breast cancer survivors are at an increased risk for osteoporotic fractures not explained by lower BMD: A retrospective analysis. *N P J. Breast Cancer*. **1**, 15010 (2015). 10.1038/npjbcancer.2015.10 PMC5515201 28721367

[33] Lo, J. C. et al. Description of major osteoporotic fractures in women with invasive breast cancer who received endocrine therapy. *J. M Netw. Open.* **4**, e2133861 (2021). 10.1001/jamanetworkopen.2021.33861 PMC8600383 34787662

[34] Chen, Z. et al. Fracture risk among breast cancer survivors: results from the Women’s Health Initiative Observational Study. *Arch. Intern. Med.* **165**, 552–558 (2005). 15767532 10.1001/archinte.165.5.552

[35] Neuner, J. M. et al. Fractures in a nationwide population-based cohort of users of breast cancer hormonal therapy. *J. Cancer Surviv*. **12**, 268–275 (2018). 29243101 10.1007/s11764-017-0666-4

[36] Hui, S. K. et al. Spatial and temporal fracture pattern in breast and gynecologic cancer survivors. *J. Cancer*. **6**, 66–69 (2015). 25553090 10.7150/jca.10288 PMC4278916

[37] Schmeler, K. M. et al. Pelvic fractures after radiotherapy for cervical cancer: Implications for survivors. *Cancer* **116**, 625–630 (2010). 20052724 10.1002/cncr.24811 PMC5084848

[38] Vogt, A. et al. Multiple primary tumours: Challenges and approaches, a review. *E S M O Open.* **2**, e000172 (2017). 10.1136/esmoopen-2017-000172 PMC5519797 28761745

[39] Kelsey, J. L., Browner, W. S., Seeley, D. G., Nevitt, M. C. & Cummings, S. R. Risk factors for fractures of the distal forearm and proximal humerus. The Study of Osteoporotic Fractures Research Group. *Am. J. Epidemiol.* **135**, 477–489 (1992). 1570814 10.1093/oxfordjournals.aje.a116314

[40] Christiansen, B. A. & Bouxsein, M. L. Biomechanics of vertebral fractures and the vertebral fracture cascade. *Curr. Osteoporos. Rep.* **8**, 198–204 (2010). 20838942 10.1007/s11914-010-0031-2

[41] American Cancer Society. Finding Bone Metastases. Available from: https://www.cancer.org/treatment/understanding-your-diagnosis/advanced-cancer/finding-bone-metastases.html. [Accessed 11 September 2025].

[42] Hamajima, N. The Japan Multi-Institutional Collaborative Cohort Study (J-MICC Study) to detect gene-environment interactions for cancer. *Asian Pac. J. Cancer Prev.* **8**, 317–323 (2007). 17696755

[43] Hara, M. et al. Factors associated with non-participation in a face-to-face second survey conducted 5 years after the baseline survey. *J. Epidemiol.* **25**, 117–125 (2015). 25400077 10.2188/jea.JE20140116 PMC4310872

[44] Takeuchi, K. et al. Study profile of the Japan Multi-institutional Collaborative Cohort (J-MICC) study. *J. Epidemiol.* **31**, 660–668 (2021). 32963210 10.2188/jea.JE20200147 PMC8593573

[45] Kobayashi, T. et al. Validation of self-reported fractures in a follow-up survey of the Japan Multi-Institutional Collaborative Cohort study. *Ann. Clin. Epidemiol.* Preprint at (2025). 10.37737/ace.26009 10.37737/ace.26009 PMC13482525 42614596

[46] Elm, E. et al. The Strengthening the Reporting of Observational Studies in Epidemiology (STROBE) statement: Guidelines for reporting observational studies. *J. Clin. Epidemiol.* **61**, 344–349 (2008). 18313558 10.1016/j.jclinepi.2007.11.008

[47] Hippisley-Cox, J. & Coupland, C. Predicting risk of osteoporotic fracture in men and women in England and Wales: Prospective derivation and validation of QFractureScores. *B M J.* **339**, b4229 (2009). 10.1136/bmj.b4229 PMC2779855 19926696

[48] Tokudome, Y. et al. Relative validity of a short food frequency questionnaire for assessing nutrient intake versus three-day weighed diet records in middle-aged Japanese. *J. Epidemiol.* **15**, 135–145 (2005). 16141632 10.2188/jea.15.135 PMC7851066

[49] Science and Technology Agency. *Standard tables of food composition in Japan* 5th edn (Printing Bureau of the Ministry of Finance, 2000). (In Japanese).

[50] Kikuchi, H. et al. Intensity-specific validity and reliability of the Japan Public Health Center-based prospective study-physical activity questionnaire. *Prev. Med. Rep.* **20**, 101169 (2020). 32884896 10.1016/j.pmedr.2020.101169 PMC7452103

[51] Latouche, A., Porcher, R. & Chevret, S. Sample size formula for proportional hazards modelling of competing risks. *Stat. Med.* **23**, 3263–3274 (2004). 15490425 10.1002/sim.1915

[52] Ng, J. S. & Chin, K. Y. Potential mechanisms linking psychological stress to bone health. *Int. J. Med. Sci.* **18**, 604–614 (2021). 33437195 10.7150/ijms.50680 PMC7797546

[53] Yamamoto, M., Rashid, O. M. & Wong, J. Surgical management of gastric cancer: The East vs. West perspective. *J. Gastrointest. Oncol.* **6** (1), 79–88 (2015). 25642341 10.3978/j.issn.2078-6891.2014.097 PMC4294827

[54] Cortés-Ibáñez, F. O. et al. The validity of self-reported cancer in a population-based cohort compared to that in formally registered sources. *Cancer Epidemiol.* **81**, 102268 (2022). 36270059 10.1016/j.canep.2022.102268

