# Supplementary material for: Japanese cancer survivors have a higher risk of fragility fractures over ten years
Source: Sci Rep. 2026 Mar 23;16:14566. doi: 10.1038/s41598-026-45389-1 (PMC13153317; doi:10.1038/s41598-026-45389-1)
Supplement: Supplementary file 1 — Supplementary Material 1 [file 41598_2026_45389_MOESM1_ESM.docx]

| **Supplementary Table 1.** Number of cancer diagnoses and follow-up duration by cancer type (*n*=1253). | | |
| --- | --- | --- |
| Cancer diagnosis | *n* | Follow-up duration, years |
| Stomach cancer | 227 | 8.6 (3.4, 10.0) |
| Colon and rectum cancer | 197 | 7.4 (3.1, 10.0) |
| Thyroid cancer | 25 | 10.0 (7.3, 10.0) |
| Bladder cancer | 43 | 6.0 (2.0, 9.9) |
| Kidney cancer | 30 | 5.4 (1.4, 9.6) |
| Hematologic cancer | 15 | 9.9 (7.0, 10.0) |
| Breast cancer | 217 | 8.1 (3.9, 10.0) |
| Gynecological cancer | 139 | 9.9 (7.3, 10.0) |
| Prostate cancer | 111 | 5.4 (2.3, 7.2) |
| The other cancers | 249 | 5.0 (1.7, 9.3) |

Cancer diagnoses include those reported at baseline (513 individuals) and those newly identified during follow-up (740 individuals); because some participants had multiple primary cancers, the total number of cancer diagnoses exceeds the number of individuals with cancer.

Continuous values are shown as medians (interquartile range).
